# Age Is a Greater Influence on Small Saccades Than Target Size in Normal Subjects on the Horizontal Video Head Impulse Test

**DOI:** 10.3389/fneur.2019.00328

**Published:** 2019-04-16

**Authors:** David R. Jay, Debbie Cane, Simon Howe

**Affiliations:** ^1^Manchester Head and Neck Centre, Manchester University NHS Foundation Trust, Manchester, United Kingdom; ^2^Manchester Centre for Audiology and Deafness, The University of Manchester, Manchester, United Kingdom; ^3^Department of Audiology, The James Cook University Hospital, South Tees Hospitals NHS Foundation Trust, Middlesbrough, United Kingdom

**Keywords:** video head impulse, vHIT, saccades, target size, vestibular, age

## Abstract

**Objective:** This study sought to investigate whether the size of the target used in the horizontal vHIT has an effect on the saccade profile of healthy subjects, and to expand upon previous work linking age to the existence of small vHIT saccades.

**Methods:** Forty eight participants were recruited between 18 and 77 years of age, with no history of vestibular, oculomotor or neurological conditions and a visual acuity of at least 0.3 LogMAR. Participants underwent four consecutive horizontal vHIT trials using the standard target size and three smaller targets. VOR gain and metrics for saccadic incidence, peak eye velocity and latency were then extracted from results.

**Results:** Target size was a statistically significant influence on saccade metrics. As target size increased, saccadic incidence decreased while peak eye velocity and latency increased. However, a potential order effect was also discovered, and once this was corrected for the remaining effect of target size was small and is likely clinically insignificant. The effect of age was much stronger than target size; increasing age was strongly positively correlated with saccadic incidence and showed a medium size correlation with peak velocity, though not with saccadic latency.

**Conclusion:** While this study suggests that target size may have a statistically significant impact on the vHIT saccade profile of normal subjects, age has a greater influence on the incidence and size of small vHIT saccades.

## Introduction

The video head impulse test (vHIT) is a quantitative adaptation of the clinical head impulse test (1), and enables functional assessment of the high-frequency angular vestibulo-ocular reflex (VOR) in all three planes of head rotation (2, 3). Lightweight goggles worn by the subject detect head movement using an accelerometer and gyroscope, and a high frame rate video camera tracks eye movement using pupil detection algorithms. Passive, small amplitude, high velocity head impulses in the planes of semi-circular canal pairs assess the ability of the VOR to generate the eye movements necessary to main visual fixation on a target. Commercially available vHIT software calculates a measure of VOR gain (the ratio of eye to head velocity), which can be compared to stratified age-related normative data to diagnose vestibular dysfunction (4). vHIT can also detect and quantify “catch-up saccades” generated by a deficient VOR. The test has a number of potential clinical uses; as a complementary and additional part of the vestibular test battery (2, 5), as a potential screening tool (6, 7), and as a front-line diagnostic test in emergency medicine to aid in the differentiation of peripheral from central causes of acute vertigo (8, 9).

Early vHIT research focused predominantly on VOR gain and the presence or absence of catch-up saccades in known cases of vestibular loss. The focus of more recent work has been to examine the metrics of these pathological catch-up saccades (10–12), and how saccade profiles change over time as central compensation for a vestibular insult is established (13–15). Recent research has also suggested that an in-depth analysis of vHIT saccade metrics is as important as gain (16), and in some cases may provide a more detailed representation of VOR function than gain values alone (17, 18). However, several studies have highlighted the existence of smaller saccades in significant proportions of healthy individuals with no relevant clinical history and VOR gains within the normal range (12, 19–23). The existence of poorly defined small saccades in all vHIT results could lead to diagnostic uncertainty in the clinical environment. One study defined the threshold between pathological and non-pathological saccades as a peak eye velocity of 110°/s (12). However, there is very little other guidance in the literature to aid with the clinical interpretation of vHIT saccades that have lower peak velocities. Indeed, the clinical experience of the authors and reports from other vestibular clinics suggest that saccades with peak eye velocities <110°/s are very common, in the presence of normal and abnormal VOR gain, and in individuals with and without known vestibular dysfunction (22–25). Whilst a vHIT result of low VOR gain combined with repeatable, high peak velocity catch-up saccades is unequivocal, there can be diagnostic uncertainty associated with the presence of saccades with normal VOR gain. There is therefore a need for further investigation and understanding of vHIT saccade profiles in normal human physiology.

When considering possible influences on normal vHIT saccade profiles there are two possible explanations; that they are a result of experimental artifact, and/or that they are a feature of normal human physiology. One possible source of small vHIT saccades in individuals with normal gain and no history of vestibular dysfunction is the target itself. The vast majority of such saccades are “overt” (12, 20, 22, 26). Overt saccades can be seen with the naked eye and occur after the head has finished moving; approximately 150 ms from the start of the head impulse (5, 15). It is also after approximately this latency that the visual cortex can respond to visual targets which have moved (27–29), suggesting that the majority of small saccades in vHIT may be visually-driven. For an individual with reasonable visual acuity, the manufacturer-supplied 3 cm diameter vHIT target contains many points within it to fixate upon. Therefore, we hypothesize that such small saccades may represent refixations within this target. It is known that fixation stability is influenced by target size (30, 31), though counter-intuitively, these studies suggested that smaller targets produce more frequent and smaller amplitude saccades than larger targets. The only study to assess the effect of target size on the vHIT found no effect of target size on saccade incidence or peak velocity (32). However, this study only included saccades with peak velocities >50°/s. It is possible that this study missed any trends that might be seen when including smaller saccades, as other studies which did this subsequently showed mean peak velocities of 69.85°/s (SD 16.06) (20) and 55.5°/s (SD 16.9) (21). The primary aim of this study was therefore to investigate and understand any effect of target size on vHIT saccade metrics.

Additionally, several papers have shown correlations between age and saccade metrics; older adults with no known history of dizziness and normal horizontal vHIT gain generate more small saccades of greater peak velocity than similar younger individuals (12, 20, 22). Although several of these authors have posited reasons why the aging process might affect small saccade generation, this is still under discussion in the literature. The secondary aim of this study was to use a wide age spread of adult participants to confirm and elaborate on known correlations between age and saccade metrics.

## Materials and Methods

### Participants

Data from 48 participants (50% male) aged between 18 and 77 years with eight participants in each 10-year age group was collected and analyzed. Participants were recruited from the University of Manchester faculty and student body, and from the local population. Participants were included on the basis of mean vHIT gain within the age-dependent, ear specific normal range (4), and a visual acuity of at least 0.3 logMAR. This is the minimum aided or unaided acuity required to obtain a driving license in the UK. Exclusion criteria included any history of oculomotor, vestibular or neurological disorders, cervical spine fracture, limited range of neck movement, neck stiffness or pain, or recent use of medications known to suppress vestibular activity or affect the central nervous system. Participants were also excluded if they were not confident that they were able to accurately fixate the smallest target, despite having visual acuity that should allow resolution of this target at 1.5 m. All participants gave written informed consent, and the study was approved by the University of Manchester Research Ethics Committee (Ref: 2016-0422-554).

### Equipment

Participants were seated 1.5 m in front of the target. At this distance the target was beyond the maximum distance at which presbyopic changes in the ocular lens cause a failure of near accommodation (33), ensuring that older participants would be able to fixate the smallest targets provided they were not myopic. Targets were mounted on a white backdrop and positioned for each participant in the center of their visual field. The white backdrop was designed to fill the majority of the participant's visual field excepting the peripheries.

Each target was die-cut from non-reflective navy blue card (RGB = 0, 93, 255) into a circle; a simple shape without straight edges or corners to avoid providing multiple points of fixation. Four target sizes were used (Table 1). Size 4 was comparable in size to the complex shape provided as a fixation target with the ICS Impulse vHIT equipment. Each smaller size was then incrementally reduced by 1 cm in diameter. Size 1 (0.87 mm) represents the limits of resolution at 1.5 m for an individual with VA = 0.3 logMAR; the inclusion criteria for visual acuity.

**Table 1:** Target sizes.

	**Diameter of target (mm)**	**Visual angle at 1.5 m (arcmins)**
Size 1	0.87	2
Size 2	10.87	25
Size 3	20.87	48
Size 4	30.87	71

*Size 1 represents the limits of resolution for an individual with a visual acuity of 0.3 LogMAR, the inclusion criteria for visual acuity, and requires the same visual resolution to fixate at 1.5 m as the critical distances in 0.3 logMAR optotypes. Size 2 is recommended by Halmagyi and Curthoys. (1), while size 4 approximates the size of the target supplied by the manufacturer*.

To rule out disconjugacy and manifest squints, ocular range of movement was observed using a penlight and cover testing performed using a Romanes occluder. VA was tested with a 6 m Bailey-Lovie #4 logMAR chart displayed at 1.5 m, and calculated using a correction factor. vHIT testing was performed using a GN Otometrics ICS Impulse v3.0 device, calibrated using the manufacturer's protocol.

### Experimental Protocol

Goggles were fitted to each participant with the tightest tolerable tension to minimize goggle slippage over the hair or scalp. Testing began with the standard target size 4. The remaining three target sizes were then tested in a random order. This semi-randomized paradigm was chosen so as to address potential fatigue or practice effects, but avoid a situation where the first test was performed using a non-standard and potentially sub-optimal target. No participant removed the goggles between trials.

Horizontal head impulses were delivered by the principal researcher (DRJ) as described by Curthoys et al. (34). In brief, this involved impulsive movements of the participant's head in the yaw plane, initiated with the tester's hands placed on the vertex, avoiding the goggle strap. Impulses were delivered with high velocity (150–200°/s) and acceleration (~3,000°/s^2^), but low amplitude (10–20°). Direction, timing, and velocity were varied between impulses to ensure the stimulus was unpredictable. For each trial, 15 impulses in each direction were performed on each participant with a range of velocities between 150 and 250°/s. The goggle frame contains a high frame rate camera and accelerometer to capture eye movements and head movements, respectively, at a sampling rate of 250 Hz.

### Saccade Analysis

The open-source software HITCal (35) was used to extract impulse and saccade data from .xml files exported from the commercial vHIT software. The process of extraction was a combination of automatic and manual saccade identification, and manual artifact rejection. In total, 5569 impulses were analyzed. For each impulse, HITCal automatically identifies maximum head velocity and uses an area-under-the-curve algorithm to calculate gain, identical to the method used by the ICS impulse software itself. The software automatically identifies the majority of large saccades using a peak-picking function. Manual saccade analysis identified any remaining smaller saccades and confirmed automatic saccade identification trace-by-trace. Coding of artifactual impulses and their incidence is shown in Supplementary Material A, and this coding system is based on a discussion of common vHIT artifacts detailed by Mantokoudis et al. (36). An impulse was excluded if it contained artifact a-f, and included if it contained artifacts y or z. After applying the artifact rejection criteria to all impulses, the mean number of accepted impulses in a trial was 13.46 (SD 1.31), and no trial had fewer than 10 impulses.

Saccades were identified by their maximum peak eye velocity in °/s and latency from initiation of the head movement in milliseconds. Minimum saccade velocity was set at 20°/s to distinguish saccades from artifacts and noise. This is in line with the guidance on artifacts from Mantokoudis et al. (36) and the artifact rejection criteria used in this study's analysis (Supplementary Material A). This minimum value also compares well with previous work on saccades from the vision science literature (37, 38).

Saccades were identified if they occurred between peak head velocity and 560 ms after the beginning of the impulse; the standard window of data capture in the manufacturer's software. This limit is approximately in line with other work on the latest time point that a saccade can be attributed to an impulse which lasts an average of 250 ms (23, 39).

Saccades were classified as positive or negative depending on whether they were in the direction of VOR eye movement or opposite to it, respectively. Later analysis of negative saccades showed that their incidence was less than that of positive saccades by a factor of 6.5, and there were very few statistically significant correlations between negative saccade metrics and age, gender or VOR gain. The effects of target size and trial order seen in positive saccades were also not seen in negative saccades. The low incidence of negative saccades meant that the statistical power required to make inferences about their behavior with this sample size is limited, and therefore analysis of negative saccades is not included here.

In order to check the reliability of the combined automatic and manual saccade analysis, a randomly selected subset of 5% of all impulses was reviewed in HITCal by a second blinded analyzer (SH); choosing to accept or reject the principal researcher's saccade identification and artifact rejection. The agreement rate was 92.81%, with the few rejects being due to copy errors or differences in approach to classification of double peak artifacts. These disparities in subjective assessment were addressed, and the dataset was fully searched for similar errors and corrected.

Three mean saccade metrics were identified for leftwards and rightwards impulses: Mean incidence of saccades in a trial, mean peak velocity of all saccades in a trial and mean latency of all saccades in a trial. Saccade incidence was scaled to account for the varying number of accepted impulses in trials by dividing the incidence by the total accepted impulses in that trial and then multiplying by mean accepted impulses for all participants.

### Statistical Methods

IBM® SPSS® Statistics v 23.0 was used for all statistical analysis. Non-parametric measures were used where data violated the assumption of normality using a Shapiro-Wilks test. The majority of data on saccade metrics was not normally distributed. Spearman's Rank-Order Correlation testing was used to assess correlations between age and saccade metrics.

Friedman tests of one-way repeated measures analysis of variance by ranks were used to assess effect of target size on saccade metrics, with target size as the within-subjects factor. It was also necessary to ascertain whether there was any effect of order on saccade metrics, i.e., due to practice or fatigue effects. This was also assessed using the Friedman test with trial order as the within-subjects factor. Results were deemed statistically significant when the *p* ≤ 0.05.

## Results

### Gain and Head Velocity: Standard Target Size Only

Initially, data for the standard target size 4 was analyzed to confirm trends shown in other vHIT research. There was no significant effect of gender on vHIT gain, as previously reported (4, 20, 40). Leftward head impulse velocities were significantly faster (mean 189.6°/s, SD 24.7) than rightward velocities (mean 168.8°/s, SD 17.7) when compared using an independent samples *t*-test [*t*_(94)_ = 4.76, *p* < 0.01]; an effect of the right-handedness of the tester common to all head impulse testing (20). Rightward gain (mean 1.03, SD 0.09) was significantly higher than leftward gain (mean 0.92, SD 0.08), tested using an independent samples Mann-Whitney *U*-test (*U* = 231, *p* < 0.01). This is also a known phenomenon in vHIT related to monocular recording; gains of the adducting eye always exceed those of the abducting eye (4, 20). Data for gain was also in line with previous normative studies (4, 20, 40), and suggested that a normal range of two standard deviations is 0.85–1.21 for right ears and 0.76–1.08 for left ears.

Peak head velocity was significantly negatively correlated with age for leftwards (Pearson's *r* = −0.42, *n* = 48, *p* < 0.01) and rightwards impulses (Pearson's *r* = −0.48, *n* = 48, *p* < 0.01); a known phenomenon thought to be related to degenerative changes in the cervical spine (41, 42). Peak head velocity was also significantly negatively correlated with VOR gain for rightwards impulses [Spearman's *r*_s_ (48) = −0.30, *p* = 0.04], though not for leftwards impulses [Spearman's *r*_s_(48) = −0.27, *p* = 0.06]. A negative correlation of gain with increasing head velocity has also been shown previously (4, 19). There was initially no statistically significant correlation between age and mean VOR gain in either ear [left: *r*_s_(48) = −0.12, *p* = 0.42; right: *r*_s_(48) = 0.04, *p* = 0.78]. However, as the data showed a negative correlation between age and head velocity, and also between head velocity and gain, it was necessary to analyse age and gain data while controlling for head velocity. This analysis was undertaken using individual impulse data for both ears rather than average values for gain and head velocity, so as to avoid masking any trends by averaging the data. Using a partial bivariate correlation to control for head velocity, a weak correlation was shown between age and gain [*r*_s_(1, 249) = −0.13, *p* < 0.01]. This correlation disappeared when participants in the top two age groups (58–67 and 68–77) were removed from the analysis.

### Saccade Metrics: Standard Target Size Only

**Figure 1:**
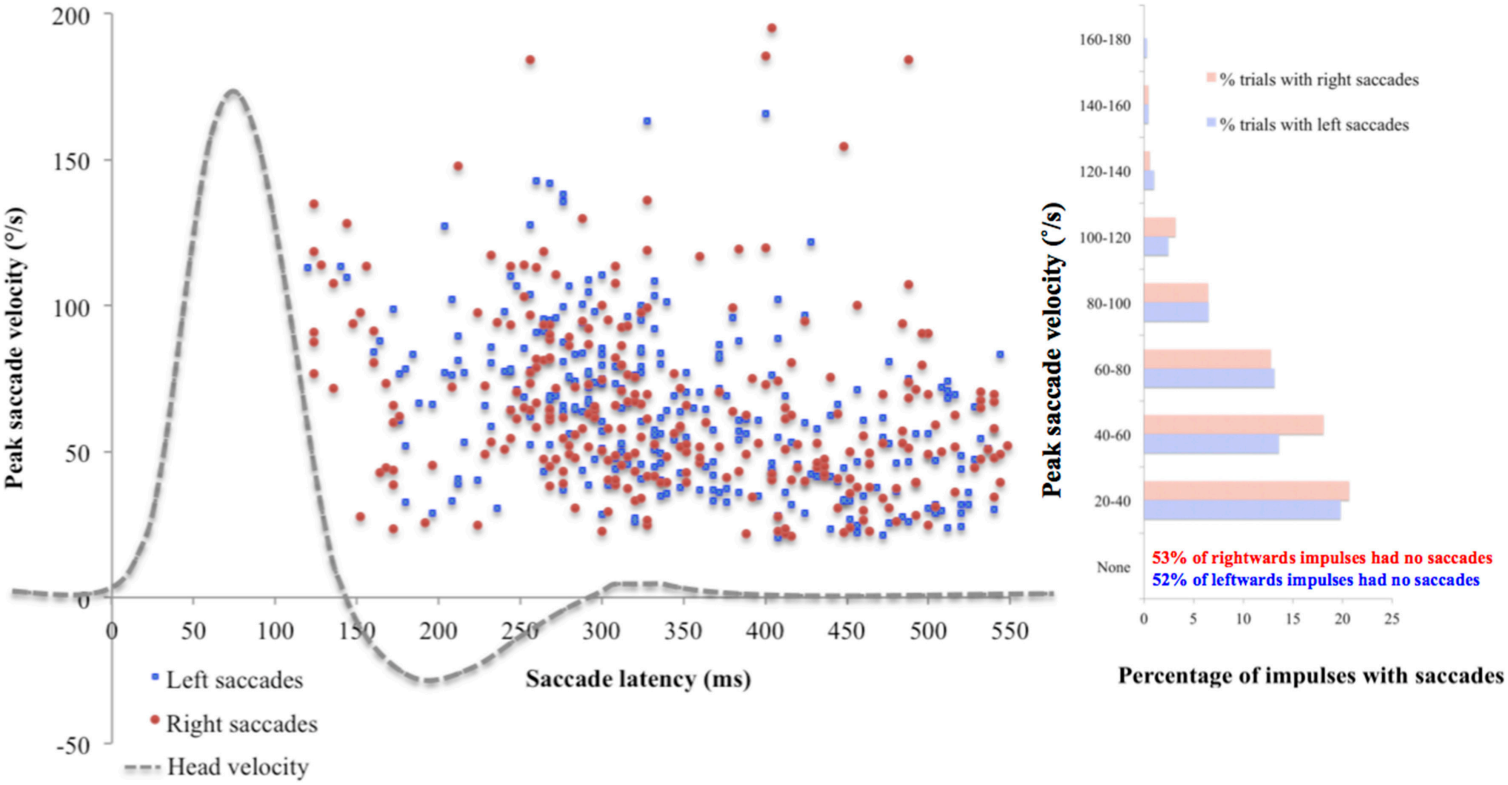
Saccade peak velocity and latency. Positive saccades produced by the whole cohort on the first trial using standard target size 4. The majority of saccades were overt (occurring after the end of the head impulse). Across the cohort, mean incidence was 5.70 (SD 4.64) saccades for left ears and 5.81 (SD 4.41) saccades for right ears, mean peak velocity was 62.94°/s (SD 24.63) for left ears and 58.63 °/s (SD 24.63) for right ears, and mean latency was 364.38 ms (SD 75.46) for left ears and 379.22 ms (SD 69.05) for right ears. There were no statistically significant differences found between ears on any saccade metric (incidence: *U* = 1114, *p* = 0.78; peak velocity: *U* = 919, *p* = 0.45; latency: *t*_(88)_ = −0.97, *p* = 0.33).

Mean saccade metrics were initially analyzed for the standard target size 4, to gain an understanding of their nature in normal physiology. None of the saccade metrics correlated significantly with VOR gain, peak head velocity or visual acuity. To illustrate the spread of the data, Figure 1 uses individual saccade data rather than mean saccade metrics, and also demonstrates that while the majority of impulses contained no saccades, many did, and in fact all trials included at least one impulse with one saccade over 20°/s peak velocity.

Differences between leftward impulses and rightward impulses in terms of these saccade metrics for target size 4 were assessed using Mann-Whitney U tests for nonparametric data and independent-samples *t*-tests for normally distributed data. There were no significant differences found between means [incidence: *U* = 1,114, *p* = 0.78; peak velocity: *U* = 919, *p* = 0.45; latency: *t*_(88)_ = −0.97, *p* = 0.33]. Therefore, a bilateral pooled mean was calculated for each saccade metric, giving each participant a measurement for each target size of:

- Their mean bilateral incidence in a single trial of positive saccades
- Their mean bilateral peak eye velocity of positive saccades in °/ s
- Their mean bilateral latency of positive saccades in seconds

**Figure 2:**
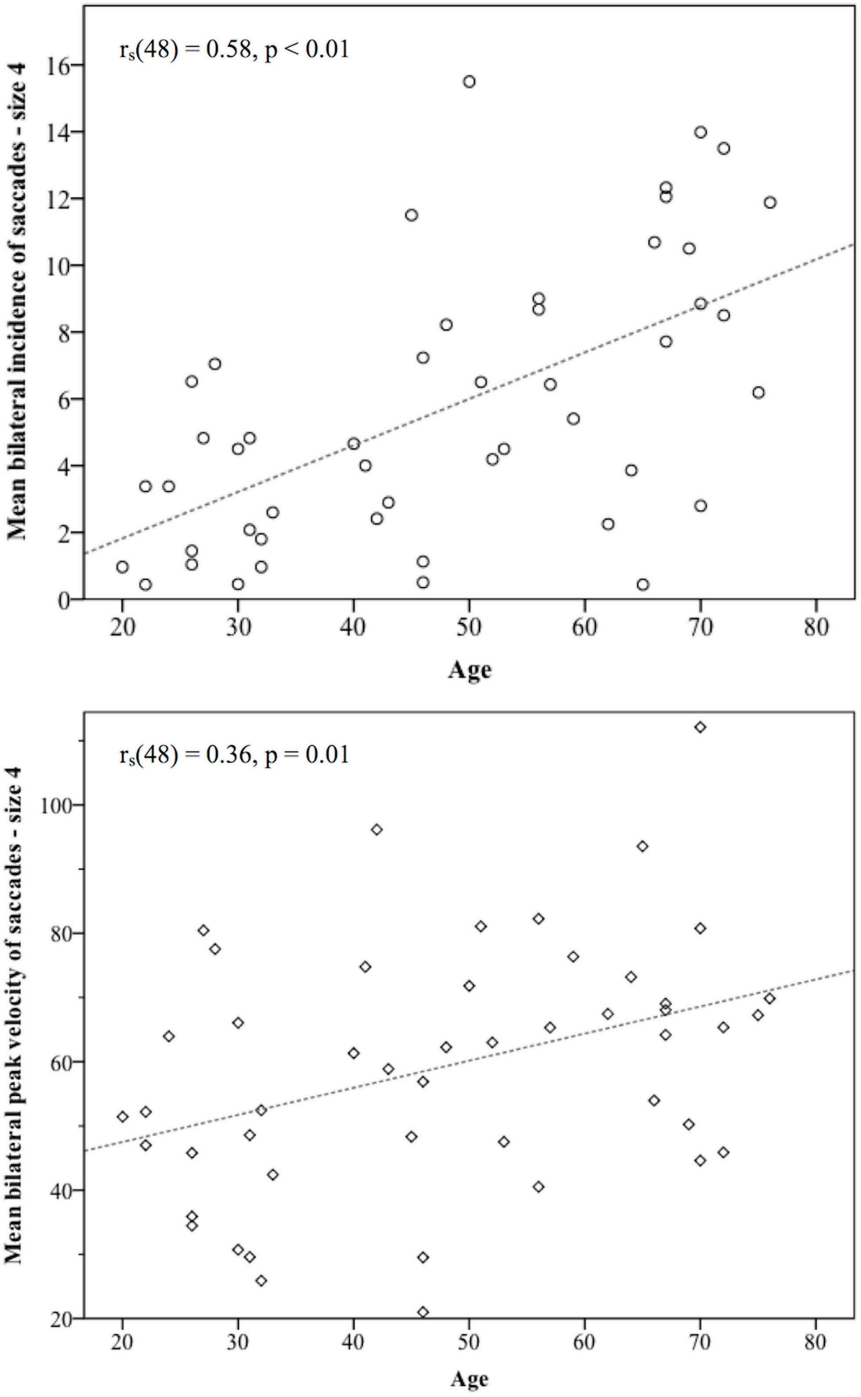
Statistically significant correlations between age and saccade metrics. Incidence of non-pathological saccades was strongly correlated with age [*r*_s_(48) = 0.58], and their peak velocities showed a medium size correlation with age *r*_s_(48) = 0.36. As participants became older, the number of saccades they produced and their size in the vHIT trace both increased.

Across the cohort, for the standard target size 4, mean bilateral incidence was 5.72 (SD 4.64) saccades, mean peak velocity was 59.31°/s (SD 19.24) and mean bilateral latency was 372.86 ms (SD 64.55) (Table 3). Age-stratified means for saccade metrics are shown in Table 2. Spearman's rank-order correlations were run to determine the relationship between age and saccade metrics. Age was significantly positively correlated with saccadic incidence [*r*_s_(48) = 0.58, *p* = 0.00] and peak velocity [*r*_s_(48) = 0.36, *p* = 0.01]. Figure 2 illustrates these correlations for the standard target size 4. There was no significant correlation between age and saccade latency [*r*_s_(48) = −0.23, *p* = 0.11].

**Table 2:** Mean bilateral saccade metrics for age.

		**Mean bilateral saccade incidence (SD)**	**Mean bilateral saccade peak velocity in °/s (SD)**	**Mean bilateral saccade latency in ms (SD)**
Age group	18–27	2.77 (2.15)	51.40 (15.03)	400.33 (59.71)
	28–37	3.03 (2.24)	46.65 (18.37)	375.05 (86.22)
	38–47	4.29 (3.60)	55.85 (23.87)	380.19 (54.98)
	48–57	7.88 (3.57)	64.22 (14.71)	356.42 (61.09)
	58–67	6.84 (4.57)	70.77 (11.36)	373.67 (79.08)
	68–77	9.52 (3.78)	67.01 (22.25)	342.65 (43.98)

Gender did have some significant effects on saccade metrics. Compared with females, males produced more saccades (*U* = 168, *p* = 0.01), with higher peak velocities (*U* = 143, *p* < 0.01) and these occurred earlier (*U* = 183, *p* = 0.03). However, as gender was only equal across the cohort and not within age groups, there were more males in some of the older age groups. As age had been shown to correlate with saccade incidence and peak velocity, a rank analysis of covariance (43) was performed to adjust for age as a covariate. Once age was taken into account, gender was only found to be statistically significant for incidence and peak velocity, but not for latency. The effect sizes were small [saccade incidence: *F*_(1, 48)_ = 4.19, *p* = 0.04, *n*^2^ = 0.08; peak velocity: *F*_(1, 48)_ = 7.94, *p* < 0.01, *n*^2^ = 0.14].

### Analysis of all Target Sizes

Mean bilateral VOR gain for each participant was first tested using a Friedman test of one-way repeated measures analysis of variance by ranks, in order to check whether target size or trial order affected VOR gain. There was no significant effect of size [$\chi_{\left(3\right)}^{2}$ = 10.65, *p* = 0.89] or trial order [$\chi_{\left(3\right)}^{2}$ = 1.83, *p* = 0.61] on gain values.

**Figure 3:**
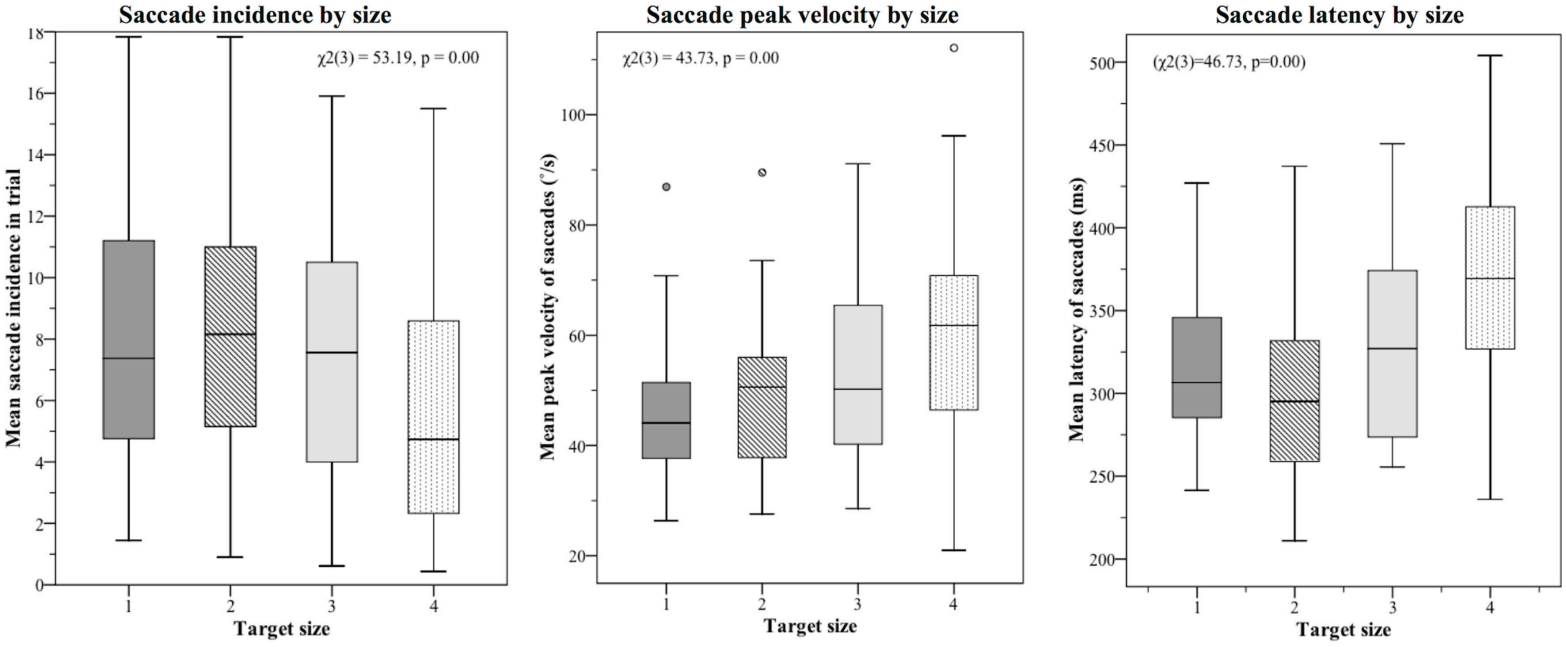
Saccade metrics plotted by target size. The largest target size 4 was always tested first, and the effect of trial order was also statistically significant. When the first target was removed from analysis, order effects became non-significant, though a small but significant effect of target size remained (effect sizes: 0.08 (incidence), 0.12 (velocity), 0.13 (latency). The small effect of target size is best summarized as such: Smaller targets produce more saccades, which tend to be smaller and occur earlier; larger targets produce fewer saccades, but these have higher peak velocities and occur later in the recording window.

Mean saccade metrics for target size and trial order are given in Table 3. All saccade metrics were analyzed using the Friedman test, performed twice using different within-subjects variables: target size and trial order (Table 4). Kendall's coefficient of concordance (W) was also calculated to approximate effect size. Increasing target size was significantly associated with decreasing incidence of saccades [$\chi_{\left(3\right)}^{2}$ = 53.19, *p* < 0.01], increasing peak velocity [$\chi_{\left(3\right)}^{2}$ = 43.73, *p* < 0.01], and increasing latency [$\chi_{\left(3\right)}^{2}$ = 46.73, *p* < 0.01] (Figure 3). Analysis using trial order as the within-subjects factor showed the reverse significant associations: when analyzed across all four trials, trial order appeared to increase incidence [$\chi_{\left(3\right)}^{2}$ = 45.55, *p* < 0.01], decrease peak velocity [$\chi_{\left(3\right)}^{2}$ = 32.52, *p* < 0.01] and decrease latency [$\chi_{\left(3\right)}^{2}$ = 37.68, *p* < 0.01]. Using separate Wilcoxon signed-rank tests between repeated measures with a Bonferroni correction to adjust for multiple comparisons (*p* < 0.017), *post-hoc* testing was conducted to investigate where the significant differences occurred; these are included in Supplementary Material B. For each saccade metric, the effect of order was always between the first trial (performed with the largest target) and the other randomized target sizes. *Post-hoc* analysis of target size also showed this difference; size 4 was always significantly different from the smaller sizes. Friedman repeated measures testing was repeated without the first trial, looking only at the randomized smaller sizes (Table 4). For all saccade metrics the effect of trial order disappeared, and while the effect of target size remained, the effect sizes were small; incidence: *W* = 0.08; peak velocity: *W* = 0.12, latency: *W* = 0.13.

**Table 3:** Mean bilateral saccade metrics for target size and trial order.

		**Mean bilateral saccade incidence (SD)**	**Mean bilateral saccade peak velocity in °/s (SD)**	**Mean bilateral saccade latency in ms (SD)**
Target size	1	8.18 (4.48)	45.65 (11.55)	315.84 (45.77)
	2	8.2 (4.29)	48.9 (12.71)	299.46 (50.08)
	3	7.51 (4.08)	52.77 (15.32)	328.84 (56.6)
Trial order	4 / 1st	5.72 (4.13)	59.31 (19.24)	372.89 (64.55)
	2nd	7.4 (4.13)	50.26 (14.58)	325.54 (53.57)
	3rd	8.03 (4.15)	49.08 (14.35)	313.16 (53.65)
	4th	8.31 (4.58)	48.03 (11.6)	305.99 (48.6)

*Standard target size 4 was always used on the first trial*.

**Table 4:** Repeated measures ANOVAs to investigate effects of target size and trial order.

**Friedman tests of one-way repeated measures analysis of variance by ranks**												
**Saccade metric**	**Mean incidence of saccades**				**Mean peak velocity of saccades**				**Mean latency of saccades**			
**Analysis**	**For all trials**		**Without first trial**		**For all trials**		**Without first trial**		**For all trials**		**Without first trial**	
**Within-subjects factor**	**Size**	**Order**	**Size**	**Order**	**Size**	**Order**	**Size**	**Order**	**Size**	**Order**	**Size**	**Order**
χ^2^	53.19	45.55	7.76	4.85	43.73	32.52	11.63	0.29	46.73	37.68	12/54	1.04
Df	3	3	2	2	3	3	2	2	3	3	2	2
Kendall's W	0.37	0.32	0.08	0.05	0.30	0.23	0.12	<0.01	0.32	0.26	0.13	0.01
Asymptotic significance (p)	<0.01^***^	<0.01^***^	0.02^***^	0.09	<0.01^***^	<0.01^***^	<0.01^***^	0.86	<0.01^***^	<0.01^***^	<0.01^***^	0.59

The first trial was always conducted with the largest target size 4. The order effect seen here disappears when the first trial is excluded from the analysis, and though target size remains statistically significant for all three saccade metrics, the effect size (shown by Kendall's W) is very small. Significant results (p ≤ 0.05) indicated with ^***^.

## Discussion

### Saccade Metrics

Saccade incidence was much higher in this study than others studying similar small saccades. 100% of participants had at least one impulse containing a saccade for target size 4 compared with 24.5% of 212 participants (20) and 30% of 899 participants (12). This is likely due to the low threshold for saccade peak velocity in this study (20°/s) compared with the 50°/s used by Matino-Soler et al. (20) and the 30°/s used by Rambold (12). Anson et al. included saccades with eye accelerations >4,000°/s^2^ and found 93% of 486 trials contained at least one saccade (22). The present study also used the default clinical time window of 560 ms after peak head velocity, similar to Anson et al. (22), and greater than the Rambold study which used 400 ms and yielded fewer saccades (12). Many other vHIT saccade studies do not specify minimum peak velocity or length of sample window.

Given the lack of any history of dizziness or imbalance in the participants of the present study, and the lack of suspicion of vestibular pathology; all small saccades studied here could be referred to as “non-pathological” saccades. Some studies have suggested a potential “cut-off” value to differentiate non-pathological from pathological vHIT saccades; 110°/s (12) and 136°/s (16). The mean value for bilateral saccade peak velocity across the present study's cohort for the standard target size 4 was 59.31°/s (SD 19.24). Two standard deviations from this value gives us an upper limit for the normal range of 97.79°/s. However, as older participants on average made saccades with higher peak velocities, an upper limit of the normal range for those aged between 68 and 77 years of age is actually 110.51°/s, agreeing with the findings of Rambold (12). This study illustrates how common small saccades are in vHIT, especially in older individuals, and suggests that care should be taken during interpretation of vHIT saccade profiles so as to avoid false positive results, especially when small saccades are seen in traces that are also prone to sources of error that may lower gain. The findings of small but statistically significant increases in saccade incidence and peak velocity in male participants are difficult to explain, and to the best of our knowledge, are novel findings.

Saccades are often classified as “covert” or “overt” depending on whether they occur during or after the head impulse (19). This can be quantified by identifying whether they occur before or after the head velocity crosses the y-axis for the first time, though this was not performed on a saccade-by-saccade basis in this study. The mean latency at which the head velocity trace crosses the y-axis in this study was 144.43 ms (SD 21.78). Estimating the incidence of covert saccades using this value shows that only 42 of the total 3,631 saccades recorded in the study were covert (1.16%). This is much lower than the 23% found in the study by Rambold (12), or by Anson et al. (22). Covert saccade incidence may have been affected by the classification of 11% of impulses containing the y artifact (double peak); other studies may have classified the second peak as an early covert saccade. It is arguably preferable to be conservative in the classification of these double peaks as they are not likely to be true covert saccades. Suggestions in recent literature are that double peaks may occur as a result of corneal reflections or the eyelid momentarily diverting the pupil tracker (5, 12), a ”mini-blink” without pupil tracking loss (36), or even a result of goggle slippage in the absence of the typical “eye leading the head” signature (44). If any of these explanations are correct, the second of the two peaks is not a true covert saccade.

### Age

This study showed that when head velocity was controlled for, there was a small but significant effect of age on VOR gain in the age groups 58–67 and 68–77. VOR gain declines have previously only been shown to occur in the over-70 s (20, 40, 45). The reasoning for controlling for head velocity was as follows: The data suggested that increasing age makes the performance of high velocity impulses more difficult, likely due to degenerative changes in the cervical spine (20, 41, 42), and also confirmed previous findings of higher velocity impulses tending to lower gains for all ages (4, 19, 20). Therefore, the inability to perform the higher velocity impulses in the older population may relatively raise the overall mean gains of older participants, masking the full tendency for gain to decrease with age when compared to younger subjects. Controlling for head velocity addresses this. When uncovered, this earlier decline in aVOR gain is a novel finding that may reflect the well-known effect of the aging process on the vestibular system occurring sooner than previously thought (46, 47).

**Figure 4:**
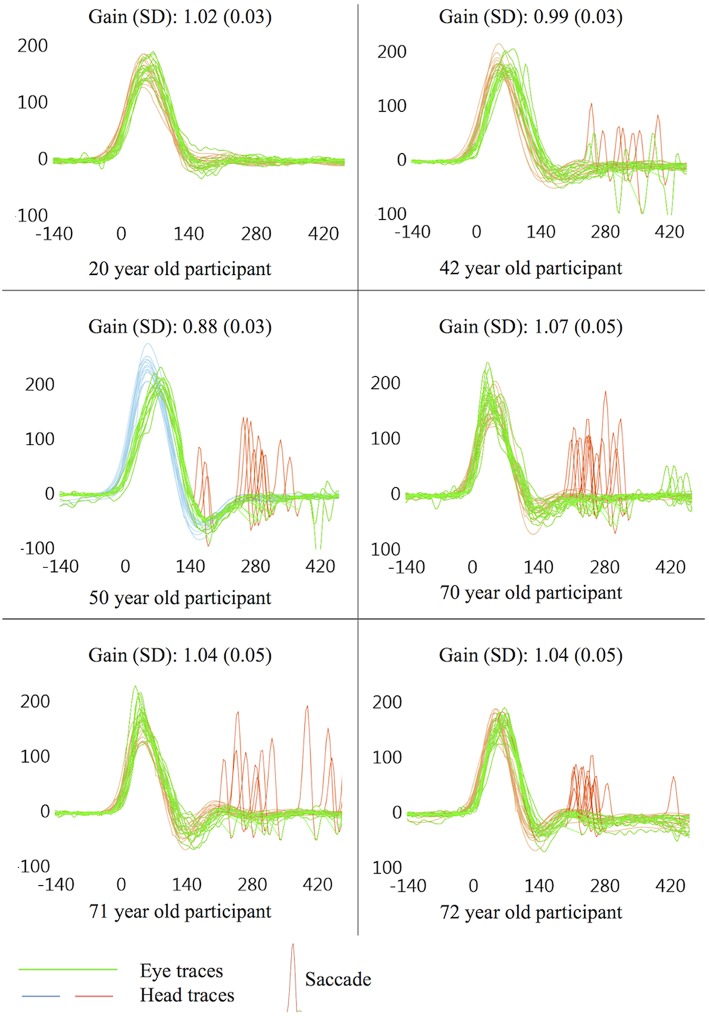
Example vHIT traces. All examples taken from a single ear on the first trial (size 4), presented here to illustrate potential findings in symptomatically “normal” individuals. Non-pathological saccades are common in vHIT, especially in older individuals, and care should be taken during interpretation of vHIT saccade profiles so as to avoid false positive results, especially when non-pathological saccades are seen in traces that are also prone to sources of error which may lower gain.

This study found a significant trend for increasing age to produce greater numbers of saccades with higher peak eye velocities, though saccade latency was not affected by age. Several other studies have shown age to be highly correlated with vHIT saccade incidence and velocity (12, 20, 22), though a more recent study which only identified saccades >50°/s only found a weak correlation with saccadic incidence and none with saccade peak velocity (16). Given the strength of the correlations shown here [*r*_s_(48) = 0.58 for incidence and *r*_s_(48) = 0.36 for peak velocity], age appears to have a much stronger effect on saccade profiles than the weak effects of target size discussed below, and is likely to be a greater influence on small saccades in vHIT. Figure 4 illustrates typical vHIT traces of individuals of different ages. These examples illustrate the difficulties facing clinicians in interpreting vHIT saccade profiles, particularly in older individuals.

The cause of age-related small saccades is still being debated in the literature. Rambold (12) suggests that the most likely cause is a declining ability to suppress reflexive saccades to the goggle frame or nose as they enter the visual periphery during an impulse (48, 49). However, this theory does not provide an entirely satisfactory explanation for the trend of increasing saccade peak velocities with age. Due to the linear main sequence relationship, greater peak velocities imply larger angles covered by saccades. If the new object entering the visual field is at approximately the same distance for all participants, saccade velocities should not increase with age, only incidence. It is not clear how this theory explains increasingly larger saccades to the same peripheral object as age increases.

One suggestion from several authors is that such saccades are compensating for a natural decline in VOR function, which might manifest itself as small reductions in gain within the normal range. While it is well established in the literature that a sub-normal VOR gain due to vestibular hypofunction is well correlated with pathological catch-up saccade incidence (19, 50), velocity and latency (51, 52), the only two studies to suggest that this relationship may exist when gains decline within the normal range are by Anson et al. (22, 23). However, correlations between gain and saccade metrics in these studies were only present in older groups, which may explain why there were no such correlations found in the analysis for the present study, which looked at gain across the whole cohort (18–77 years). Also, correlations between age and saccade metrics in the present study began at a much younger age than the small effect of age on VOR gain found once head velocity was controlled for. Indeed Anson et al. (22) found that older individuals make larger and more frequent saccades even after controlling for small changes in gain, indicating that an increase in incidence and velocity of saccades with age is insufficiently explained by an aging VOR lowering gain and generating retinal slip.

Voluntary saccadic latency is known to increase with age (39, 53), as do motor reaction times on other tasks in older age groups (54). However, these studies characterized voluntary saccades to acquire new targets, rather than the involuntary saccades seen in this and other studies (20, 21, 40). In this study, latency of saccades did not show a significant correlation with age, agreeing with the findings of Anson et al. (22) who suggest that this may be explained by considering such saccades as a compensatory solution to age-related degeneration of peripheral or central vestibular function. First we must consider cases of unilateral vestibular loss; it is known that catch-up saccades are a learned behavior to correct for retinal slip (55), and that these tend to reduce in size, latency and variability as compensation occurs (52, 56). Therefore, if the sub-110°/s saccades seen here and in other studies do represent age-related vestibular degeneration, participants may have been captured at many different stages of compensation for subtle and gradual bilateral vestibular loss, thus masking any patterns in saccade latency across the cohort compared with those that might be seen longitudinally within one individual. Small saccades such as those found in this and other studies may be subtle clues representing aging of the central or peripheral vestibular system that is not reflected in gross changes in VOR gain (22, 23, 26).

### Size Effects

Initially there appeared to be a significant effect of both size and order on saccade metrics. *Post-hoc* analysis revealed that the first trial using the largest target (size 4) was the cause of the order effect. When this trial was removed from analysis, order effects became non-significant, though a small but significant effect of target size remained (Table 4). Therefore, it is likely that the effect of trial order is merely reflecting the larger size of the first target, though due to the experimental protocol it is impossible to completely rule out a small first-test only practice effect in addition to the effect of target size. Once we set aside the largest target, the statistically significant effect of size is small; *W* = 0.08 (incidence), *W* = 0.12 (velocity), *W* = 0.13 (latency), which is likely why no such effect of size was seen in a recent study that only included saccades with peak velocities >50°/s (32).

The small effect of target size is best summarized as such: Smaller targets produce more saccades, which tend to be smaller and occur earlier; larger targets produce fewer saccades, but these have higher peak velocities and occur later in the recording window. The effect of target size on saccade peak velocity is not well explained by the original hypothesis of the authors; that due to the greater possibilities for fixation available within each larger target there is more room for the eye to move within it. As target size in this study increased, so did mean saccade peak velocity. The targets were incrementally 1 cm bigger, each offering an additional 23 arcminutes (0.38°) of visual angle inside the target for each size increment. When considering saccades, peak velocity is related linearly to angular distance by the “main sequence” relationship (57). Therefore, increases and decreases in peak eye velocity can also be thought of as increases and decreases in angular distance; essentially the two metrics are interchangeable. In this study, while target size increased linearly, the peak eye velocity did not, with differences between means generally becoming larger for each increase in target size (Table 3). If the saccadic movements were entirely related to refixations within the target, the velocity of saccades might be expected to increase at a constant rate with the target size. Furthermore, incidence of saccades decreased for larger targets, inconsistent with the theory of refixations within the target.

An alternative theory based on gaze fixation stability (58), addresses not only the tendency for peak velocity to increase with larger targets, but also the significant effect of size on saccade incidence (larger targets, fewer saccades) and saccade latency (larger targets, later saccades). All three of these relationships are seen in a study by McCamy et al. (31), which examined microsaccades with similar peak velocities produced when healthy adult subjects were required to fixate targets of various sizes. As target size increased, saccade incidence decreased, saccade amplitude (peak velocity) increased and inter-saccade interval (latency) increased. McCamy et al. (31) suggest two complimentary reasons for this, the simpler of the two being that subjects tend to relax their fixation for larger targets, making fewer saccades as long as they feel that their gaze is held somewhere inside the target. While this theory could explain a reduction in saccade numbers with larger targets, it does not address the increases seen in peak velocity or latency. The second explanation builds on the suggestions of Timberlake et al. (59) and relates target size to the fovea centralis; the portion of the retina with the highest visual acuity and spatial resolution, covering the central 1.5–2° of the visual field. McCamy et al. (31) suggest that targets small enough to fit entirely within the fovea of the retina produce more frequent, accurate and faster retinal error signals than targets that extend out beyond this area. An error signal is produced when the image of the target on the retina moves from the desired fixation point due to small movements of the head or eye. The size of such a signal is the distance of the target on the retina from the center of the fovea (60). Larger targets may have edges outside the area where error detection is of the highest resolution, producing less accurate error signals and leading to a decrease in the number of saccades generated to correct for the error, an increase in latency due to the decreasing error detection ability, and producing larger saccades due to the greater distances needed to refixate. While the targets in McCamy et al. and in this study are all small enough to fit inside the 1.5–2° (90–120 arcminutes) of the fovea, the framework suggested by the authors may translate down to sub-foveal structures where cone-spacing, visual acuity (and by extension, error detection) have been shown to steadily increase into the foveal center (61). The smallest targets therefore may produce more numerous, smaller and earlier saccades because the target falls on the retina entirely in an area which is more likely to detect whether they are being properly fixated.

This theory might therefore suggest that the optimum target size for vHIT is a larger target. However, saccades which are generated for larger targets do tend to have higher peak eye velocities and potentially could be more easily confused with pathological catch-up saccades, leading to false positive vHIT results. Target size 3 (approximately 2 cm diameter) may represent a good compromise between saccade incidence and size of saccades, avoiding the numerous saccades of smaller targets and the larger peak velocities of saccades seen on the current standard target. This is line with the recommendation of Curthoys et al. (34) with respect to target size. Clinicians performing vHIT should be aware of the trends identified in this study and bear them in mind when interpreting vHIT results. However, it should also be noted that while the effect of target size on saccade metrics is statistically significant, the effect size is of a magnitude that may be insignificant in clinical practice, and arguably has a minor effect on vHIT results in comparison with age, and certainly when compared with other common sources of error such as goggle slippage, unreliable pupil tracking and poor patient cooperation. No alternative target sizes reduced the incidence of small saccades to zero, and this study suggests that not only is the target itself not the sole cause of small saccades, but that vHIT can be performed with a number of different sized targets without grossly affecting the saccade profile in a clinical context.

## Limitations

The decision to use the standard target size for the first trial and follow with randomized smaller sizes was taken so as to establish the standard protocol for the participant before attempting alternatives, and to avoid the possibility of the participant's first trial being undertaken with a small, non-standard target. The unfortunate consequence of this is that it is impossible to completely separate the size effect from a potential order effect, and so judgements on the relative magnitude of these effects are clouded by their interaction in target size 4. An alternative approach in future studies to rule out any potential order effect could be to use a practice trial to diminish any effects of a first trial before carrying out the experimental trials, or to also use a larger target to track any size effects above size 4.

The weak correlations and small effect sizes seen here may reflect the small sample size; specifically for the correlation between age and VOR gain (once head velocity is controlled for), and for the size effect itself. Larger studies may be more effective in corroborating these findings, particularly in confirming subtle, hidden declines in VOR gain and any relationship this “corrected” value for gain may have with small saccades.

One major limitation of this study is the lack of a metric for variance around the mean latency for each trial, and hence a measure of how gathered or scattered the saccades were, as described in other studies (14, 20, 35). This metric could use standard deviation calculated from individual saccades for each trial, or coefficient of variation as used in HITCal's PR metric for saccade organization, though this was not recorded in this study. Future work in this area could attempt to calculate such a metric for variance and apply to it the same statistical tests as for other saccade metrics. This may show interesting trends in how saccade dispersion changes in relation to age, trial order and size.

It is a methodological limitation of this study that due to the 6 m LogMAR chart used with a correction factor, VA <0.1 could not be recorded, therefore limiting the ability of such analysis to fully assess any correlations with the best visual acuities.

## Conclusions

This study has shown a small but significant effect of target size on the incidence, peak velocity and latency of small saccades in the horizontal vHIT. The study also found a weak but significant correlation between age and VOR gain in the over-58 age categories. Most significantly, the study confirmed and further developed the links shown by other authors between age and the saccade profile of normal subjects on the horizontal vHIT. The data showed strong correlations between age and saccadic incidence and velocity, which may suggest that small vHIT saccades represent bilateral age-related vestibular deterioration that is not reflected by gross changes in VOR gain.

## Ethics Statement

This study was carried out in accordance with the recommendations of Guidance for research ethics published online by the University of Manchester Research Ethics Committee, with written informed consent from all subjects. All subjects gave written informed consent in accordance with the Declaration of Helsinki. The protocol was approved by the University of Manchester Research Ethics Committee (Ref: 2016-0422-554).

## Author Contributions

DJ designed the experiment with input from SH and DC. DJ collected the data, drafted the manuscript, conducted the statistical analysis and interpreted the statistical analysis. SH performed secondary assessment of data to check inter-rater reliability. DJ, SH, and DC critically edited the manuscript. All authors approved the submitted version of the manuscript and are accountable for the accuracy and integrity of the work.

### Conflict of Interest Statement

The authors declare that the research was conducted in the absence of any commercial or financial relationships that could be construed as a potential conflict of interest.
