# Supplementary material for: Age Is a Greater Influence on Small Saccades Than Target Size in Normal Subjects on the Horizontal Video Head Impulse Test
Source: Front Neurol. 2019 Apr 16;10:328. doi: 10.3389/fneur.2019.00328 (PMC6476940; doi:10.3389/fneur.2019.00328)
Supplement: Supplementary file 1 [file Data_Sheet_1.docx]

| **Category** | **Reason** | **Limits** | **N** | **% of total impulses** |
| --- | --- | --- | --- | --- |
| a | **Phase shift** Eye leads head (goggle slippage) | Eye trace 20ms in front of head trace on x axis. Sustained for at least 30ms in first half of head peak (between -8ms and 52ms) | 0 | 0.00% |
| b | **Noise / pupil detection error** Continuous oscillations around baseline | Oscillations larger than 20°/s peak to peak lasting longer than 150ms in period between 52ms and 560ms | 19 | 0.34% |
| c | **Eye movement pre-stimulus** (when head is still) | Eye deviates from head trace by more than 20°/s in either direction in the 50ms before start of impulse. Indicates potentially compromised initiation of VOR. Ignore synchronised head/eye movement that is true impulse/VOR. | 131 | 2.35% |
| d | **Head movement pre-stimulus** (eye follows) | Faster than 20°/s in either direction in the 50ms before start of impulse. Indicates potentially compromised initiation of VOR. Ignore synchronised head/eye movement that is true impulse/VOR. | 43 | 0.77% |
| e | **Blinks** | Peaks in eye trace with high amplitude (≥75% of peak eye trace)  AND short duration; ≤150ms  AND crosses head velocity baseline by more than 20°/s | 25 | 0.45% |
| f | **Mini-blink** during first half of impulse | An eye movement in the direction of the head movement which occurs during the first half of the head impulse and is greater than 20°/s. Indicates potentially poor initiation of VOR. | 172 | 3.09% |
|  | | **Total impulses excluded** | **412** | **7.40%** |
|  |  |  |  |  |
|  | **Reason** | **Limits** |  |  |
| y | **Double peak**  (Eyelid clipping or mini-blink during second half of impulse) | An eye movement in the direction of the head movement which occurs during the second half of the head impulse and is greater than 20°/s | 609 | 10.94% |
| z | **Rebound / overshoot** | Head velocity greater than 50°/s in opposite direction | 619 | 11.12% |
|  |  | **Impulses with no artifacts** | **3877** | **69.62%** |

**Supplementary material A.** Artifact criteria, adapted from Mantokoudis *et al.* (36). Impulses containing any artifact a-f were excluded from analysis. Artifacts y and z were included: neither were significantly correlated with aVOR gain, any saccade metric, age or visual acuity.
