# Supplementary material for: Age Is a Greater Influence on Small Saccades Than Target Size in Normal Subjects on the Horizontal Video Head Impulse Test
Source: Front Neurol. 2019 Apr 16;10:328. doi: 10.3389/fneur.2019.00328 (PMC6476940; doi:10.3389/fneur.2019.00328)
Supplement: Supplementary file 2 [file Data_Sheet_2.docx]

| **Saccade incidence by size**    **Z = -5.585, p = 0.000*****  **Z = -5.053, p = 0.000*****  Z = -2.053, p = 0.040  **Z = -5.313, p = 0.000*****  Z = -2.241, p = 0.025  Z = -0.667, p = 0.505 | **Saccade incidence by trial order**    Z = -0.804, p = 0.421  Z = -2.595, p = 0.009  Z = -1.918, p = 0.055  **Z = -5.231, p = 0.000*****  **Z = -4.783, p = 0.000*****  **Z = -4.550, p = 0.000***** |
| --- | --- |

**Supplementary material B.** Post hoc testing using separate Wilcoxon signed ranks tests with Bonferroni correction for multiple comparisons. Differences between means were deemed significant when p ≤ 0.0017, and are marked in bold with ***. For all three saccade metrics, when analysing by trial order, significant differences were only found between the first trial (largest target) and subsequent trials, whereas when analysed by target size, significant differences were found between all target sizes. This suggests that the overall effect of trial order was merely an effect of target size caused by the first trial always using the largest target.

| **Peak velocity of saccades by size**    **Z = -3.528, p = 0.000*****  **Z = -4.903, p = 0.000*****  **Z = -2.646, p = 0.008*****  **Z = -4.646, p = 0.000*****  **Z = -3.662, p = 0.000*****  Z = -2.144, p = 0.32 | **Peak velocity of saccades by order**    Z = -1.077, p = 0.282  Z = -0.677, p = 0.498  **Z = -3.887, p = 0.000*****  Z = -0.697, p = 0.486  **Z = -4.554, p = 0.000*****  **Z = -4.677, p = 0.000***** |
| --- | --- |

| **Latency of saccades by size**    **Z = -4.339, p = 0.000*****  **Z = -3.498, p = 0.000*****  Z = -1.354, p = 0.176  **Z = -3.118, p = 0.002*****  **Z = -4.954, p = 0.000*****  **Z = -5.559, p = 0.000***** | **Latency of saccades by order**    Z = -2.328, p = 0.020  Z = -0.708, p = 0.479  **Z = -4.451, p = 0.000*****  **Z = -5.180, p = 0.000*****  Z = -1.303, p = 0.193  **Z = -4.954, p = 0.000***** |
| --- | --- |
